# The Cone of Direct Gaze: A Stable Trait

**DOI:** 10.3389/fpsyg.2021.682395

**Published:** 2021-06-29

**Authors:** Janek S. Lobmaier, Branislav Savic, Thomas Baumgartner, Daria Knoch

**Affiliations:** Department of Social Neuroscience and Social Psychology, Institute of Psychology, University of Bern, Bern, Switzerland

**Keywords:** direct gaze, cone of gaze, CoDG, stability, individual differences, Bland–Altman plot

## Abstract

Direct eye gaze is a potent stimulus in social interactions and is often associated with interest and approach orientation. Yet, there is remarkable variability in the range of gaze lines that people accept as being direct. A measure that is frequently used to quantify the range of gaze angles within which an observer assumes mutual gaze is the cone of direct gaze (CoDG). While individual differences in CoDG have often been examined, studies that systematically investigate the stability of an observers' CoDG over time are scarce. In two experiments, we measured the CoDG using an established paradigm and repeated the measurement after 5 min and/or after 1 week. We found high inter-individual variation, but high agreement within participants (ICCs between 0.649 and 0.855). We conclude that the CoDG can be seen as a rather stable measure, much like a personality trait.

## Introduction

Knowing whether another person is making eye contact or not is a pivotal skill for social interactions (Argyle and Cook, 1976; Kleinke, 1986; Baron-Cohen, 1995). In human beings, eye contact usually signals approach orientation and affiliation motivation while averted gaze is associated with avoidance orientation and disinterest. By making eye contact with another person, we signal that we are attending to him or her and that we might want to start a conversation. For a potential addressee it is therefore essential to know whether he or she is being looked at or not (cf. Hamilton, 2016). As warrants such an important skill, human beings can generally detect direct gaze rather accurately (e.g., Gibson and Pick, 1963), but there is a considerable range of gaze directions wherein people feel looked at (e.g., Gamer and Hecht, 2007; Lobmaier et al., 2008; Harbort et al., 2017; Balsdon and Clifford, 2018). This lead Gamer and Hecht (2007) to use the metaphor of a cone of gaze rather than that of a ray as assumed in earlier studies (e.g., Gale and Monk, 2000; Symons et al., 2004).

The cone of direct gaze (CoDG) refers to the range of gaze directions within which a person feels looked at: the wider the CoDG, the more liberal the observer's judgement. By accepting a relatively large range of gaze directions to be making eye contact, observers avoid the cost of missing mutual gaze, which is greater than mistakenly characterizing averted gaze as direct (Langton et al., 2004). Recent studies revealed considerable individual differences in the range of gaze angles that people accept as being direct (e.g., Ewbank et al., 2009; Gamer et al., 2011; Schulze et al., 2013; Harbort et al., 2017; Gianotti et al., 2018), with some observers being more prone to assume mutual gaze than others. Efforts to explain individual variability in the width of the CoDG focused primarily on differences in social anxiety (Gamer et al., 2011; Jun et al., 2013; Harbort et al., 2017) or autistic traits (Matsuyoshi et al., 2014). These studies found that individuals suffering from social anxiety accept a wider range of gaze lines as making eye contact whereas autistic individuals have been shown to have a narrower CoDG. Healthy participants lie somewhere in between, but also showing notable between-participant variability in the width of the CoDG.

Gianotti et al. (2018) established a relation between the CoDG and Theta resting EEG in the left temporo-parietal junction (TPJ) and adjacent posterior STS. Resting EEG has been shown to be highly specific and extremely stable over time, and is therefore considered as a neural “fingerprint.” It has been widely used to reveal sources of individual differences. Task-independent baseline activation in the TPJ and posterior STS may serve as a neural marker explaining the individual variability in the CoDG. TPJ as well as the adjacent pSTS play an important role in the mentalizing system (cf. Frith and Frith, 2006; Saxe, 2006). Given that gaze direction is a fundamental stimulus for attributing mental states (e.g., Baron-Cohen, 1995; Khalid et al., 2016) it stands to reason that baseline activation in the TPJ/pSTS is associated with individual differences in the feeling of being looked at. Hence, task-independent baseline activation in the TPJ and pSTS is a promising source for explaining the individual variability in the feeling of being looked at.

Above mentioned studies imply that the CoDG can be seen as a relatively stable trait, with some people showing a wider CoDG than others. But how stable is an individual's CoDG over time? Knowing whether the width of the CoDG varies within the same person is important, if for example the CoDG is used as a diagnostic measure for clinical disorders, such as social anxiety, or if it is used to evaluate the efficacy of a therapy. In the present study, we aimed at measuring the stability of the CoDG over time in two highly controlled laboratory experiments. We used an established paradigm to measure the CoDG (cf. Gianotti et al., 2018) at baseline, after 5 min, and after 1 week. In this task, participants view a series of centrally located faces, one at a time, and indicate whether the face is making eye contact or not. Note that others have used variants of this task in which a variable number of peripherally presented faces influenced the cone of gaze (e.g., Gamer et al., 2011; Harbort et al., 2017). We assessed the stability of the CoDG over time using intra-class coefficients (ICCs) and Bland–Altman plots (Bland and Altman, 1986). Because the CoDG has been related to social anxiety disorder (SAD) and autism spectrum disorder (ASD), we screened our participants for SAD and ASD traits. If the CoDG is indeed a stable trait-like characteristic, we would expect high ICCs, and the high within-individual agreement would be graphically reflected in the Bland–Altman plots. If however the CoDG is much influenced of the current state of the observer, this should be reflected in low ICCs and accordingly, in the Bland–Altman plots.

## Experiment 1: CoDG at Baseline and After 5 min and After 1 Week

### Methods

#### Participants

Fifty-nine participants (22 men, 37 women) aged between 19 and 54 years (*M* = 25.3, *SD* = 9.71) volunteered to take part in this study. All participants had normal or corrected to normal vision. Seven participants were excluded from the analysis due to inconsistent responses recorded during the task, which precluded the estimation of Cone of Direct Gaze (CoDG). The study was approved by the local Ethics Committee. All participants gave written informed consent and were informed of their right to discontinue participation at any time. Data were collected in a single wave and then analyzed (no analyses were calculated before all participants were tested). Each participant was tested three times, once at baseline (T0), once after a 5 min break (T1), and finally after 1 week (T2).

#### Stimuli

Three-dimensional face stimuli were created using the software package FaceGen Modeler 3.5.2 (Singular Inversions Inc., 2010) which enables the generation of face stimuli with a high level of realism. Faces of four Caucasian gender-neutral avatars showing a neutral expression were generated. To ensure that the perceptual features of different face stimuli did not affect the results, the four avatars were generated by using the “genetic” tool. This tool allows to create highly similar faces with a predefined level of randomness (30%). The gaze direction of the faces was aligned with the head direction, so that nose, gaze fixation point, and virtual camera lay on the same axis. The avatar heads obtained with this procedure were then rotated in 1° steps producing 17 different viewing angles (from 1° to 8° to the left and right, and 0°).

#### Task and Procedure

After obtaining written informed consent, participants were seated comfortably in a dimly lit room and received written instructions for the gaze discrimination task.

**Figure 1:**
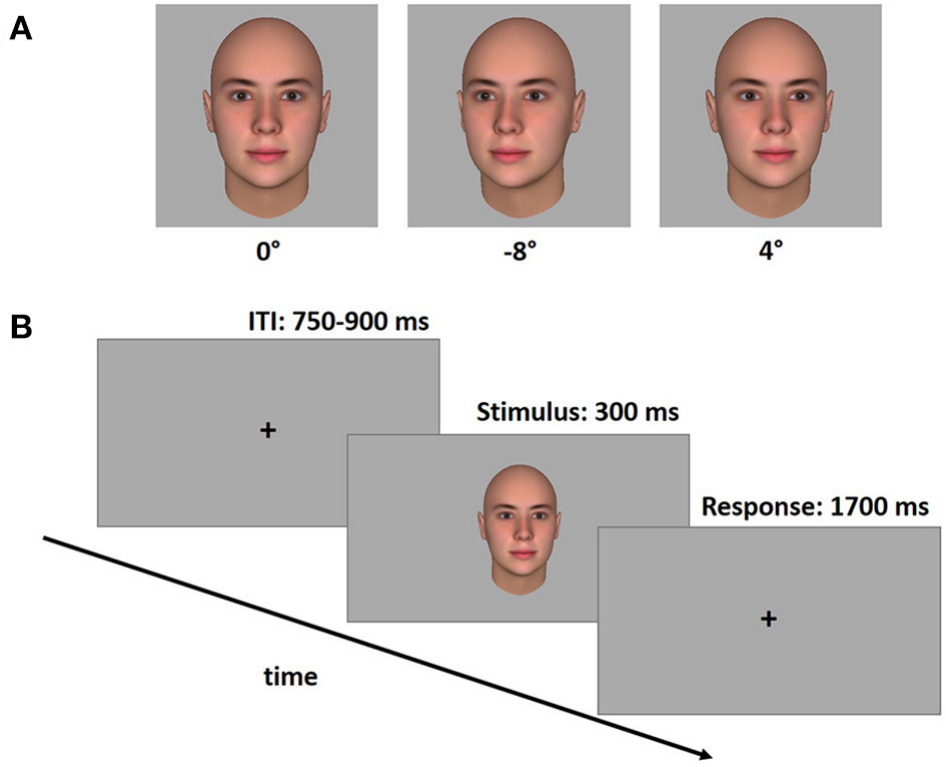
Stimulus examples in three different viewing angles **(A)** and schematic time line of the gaze discrimination task **(B)**. The task consisted of a variable inter-stimulus interval (ITI), followed by a stimulus face (300 ms), which was then replaced with a response window (1,700 ms). Participants responded whether or not the stimulus face was looking at them *via* two orthogonally arranged custom-made response buttons.

They sat at a distance of ~60 cm from a PC screen. The face stimuli appeared on the screen with a width of 6 cm, thus subtending a visual angle of ~5.7°. This corresponds to a distance of ~180 cm in real life. Lighting conditions were kept constant for all participants and the screen position was manually adapted so that the eyes of the avatars were vertically aligned with the eyes of the participants. Each face was presented for 300 ms in the center of the screen, followed by a response window of 1,700 ms, followed by an inter-trial interval (ITI) of variable duration (between 750 and 900 ms). During both the response window and the ITI period a fixation cross was shown. Participants were asked to decide as quickly as possible whether the presented face was gazing directly at them using predefined buttons on a custom made response box. A schematic timeline of the gaze discrimination task is shown in Figure 1. The keys on the response box were aligned perpendicular to each other to avoid any gaze induced response biases. The correspondence between yes/no responses, which hand was used for yes/no was counterbalanced across participants. The gaze discrimination task comprised 144 trials [18 angles (0° angle was shown twice) × 4 avatars × 2 repetitions]. Stimuli were presented pseudorandomly across three experimental blocks (48 trials each), with the constraint that each angle and face identity was equally distributed across the blocks.

One test session lasted ~10 min. The second test session took place after a 5-min break and the third test session after 1 week. All test sessions were identical, except that at the end of the third test session participants filled in the short version of the *Autism Spectrum Quotient* (AQ-k) (Baron-Cohen et al., 2001; German version by Freitag et al., 2007) and the *Social Phobia Inventory* (*SPIN*) (Connor et al., 2000). The AQ-k is a 33-item diagnostic questionnaire developed to measure the expression of Autism-Spectrum traits in an individual, by his or her own subjective self-assessment. The SPIN is a 17-item questionnaire designed as a screening instrument of social phobia. It assesses fear and avoidance of social situations as well as physiological symptoms of anxiety.

### Statistical Analyses

#### CoDG Calculation

The proportion of yes and no responses across visual angles were used to compute the CoDG. As a first step, we calculated the percentage of times the participant decided that the face stimulus was looking directly at him/her as a function of the gaze angle. We then fitted the data to a logistic function using an in-house algorithm to calculate the point of subjective equivalence (pse). The pse is defined as the angle at which a participant would be predicted to choose the yes and no responses with equal frequency (i.e., the percentage of yes and no responses equals 50%). Such analysis was conducted separately for left and right side gaze angles. The CoDG was calculated as the sum of the absolute values of the left and right side pse.

#### Stability (Test–Retest Reliability)

The stability of the CoDG over time was assessed by calculating the intraclass correlation coefficient (ICC) based on a single measurement, absolute agreement, 2-way mixed effects model (cf., Koo and Li, 2016). We first calculated the ICC over all three sessions, and then for each respective comparison (baseline vs. T1; baseline vs. T2; T1 vs. T2). Koo and Li (2016) suggest that ICC values < 0.5 are indicative of poor consistency, values between 0.5 and 0.75 indicate moderate consistency, values between 0.75 and 0.9 indicate high consistency, and values >0.90 indicate excellent consistency.

The Bland–Altman method was used to visualize the agreement between CoDG scores at baseline and after 5 min and 1 week, respectively (Bland and Altman, 1986). The Bland–Altman method uses a plot to visualize the agreement between two quantitative measurements and gauges this agreement by constructing limits of agreement (Giavarina, 2015). The limits of agreement are computed using the mean and the standard deviation of the differences between the two measurements; the upper limit of agreement = mean difference + (standard deviation of the difference × 1.96), the lower limit of agreement = mean difference–(standard deviation of the difference × 1.96), capturing the 95% CIs. The *Y*-axis of the Bland–Altman plot depicts the difference between paired measurements while the *X*-axis portrays the mean of the paired measurements. Bland–Altman plots are presented for all possible comparisons (baseline vs. T1; baseline vs. T2; T1 vs. T2). Because the later session in the comparison was always subtracted from the earlier session (e.g., baseline–5 min), positive values denote a decrease in CoDG over time, whereas negative values represent an increase of the CoDG.

#### Relation Between CoDG and Questionnaires

To assess the relationship between the CoDG and autistic traits and between the CoDG and traits of social phobia, we calculated Pearson correlations between the individual CoDG (averaged across sessions) and the AQ-k, as well as between the averaged CoDG and the SPIN values.

We also calculated a repeated measure ANOVA with session as within-participant factor and participant sex as between-participant factor. Participant age, AQ-k and SPIN values were entered as covariates. We used the Huynh–Feldt epsilon correction for heterogeneity of covariances when sphericity could not be assumed.

## Results

**Figure 2:**
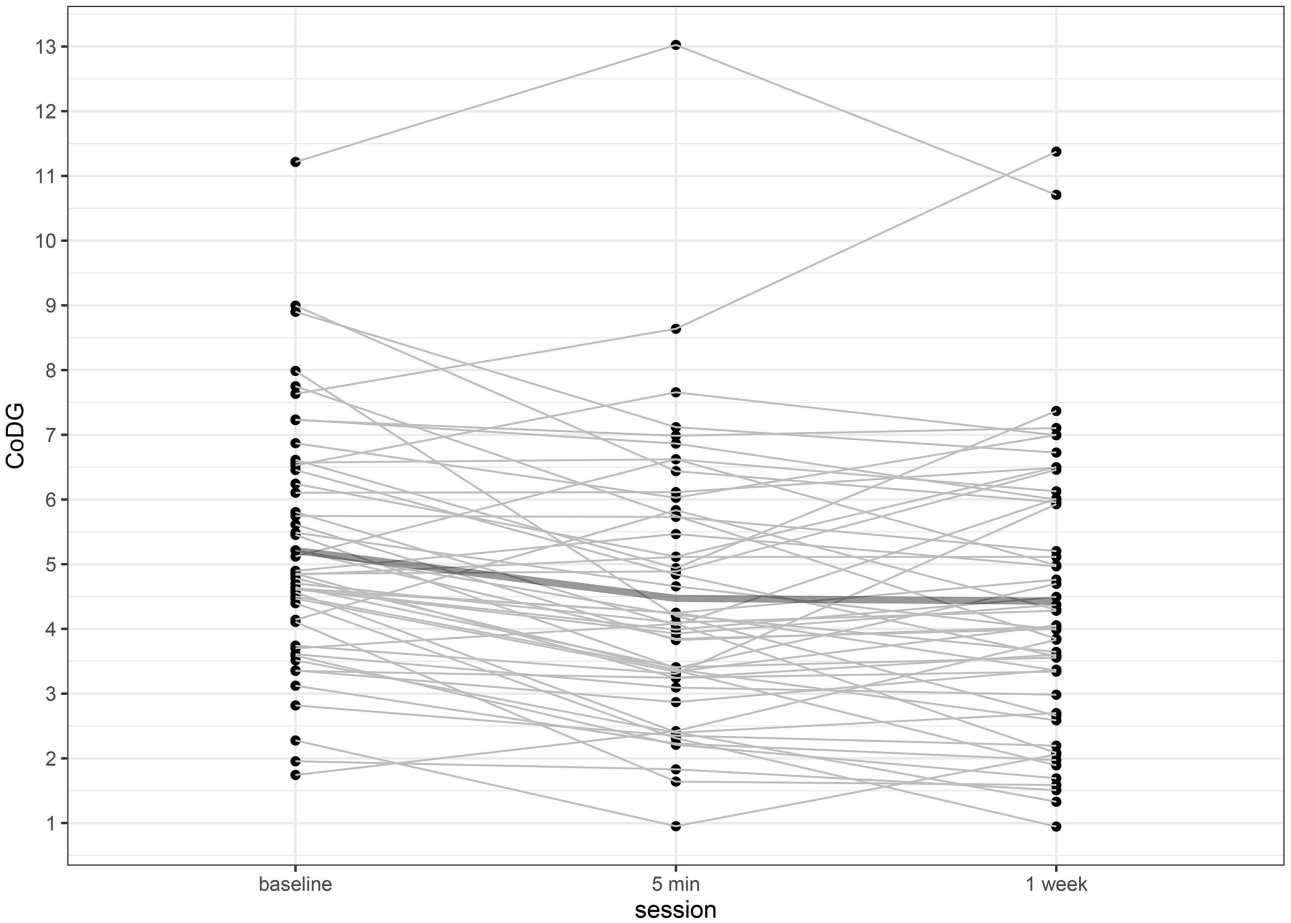
Individual CoDG in angular degrees, separately for baseline, after 5 min, and after 1 week. The bold line depicts the mean CoDG.

**Figure 3:**
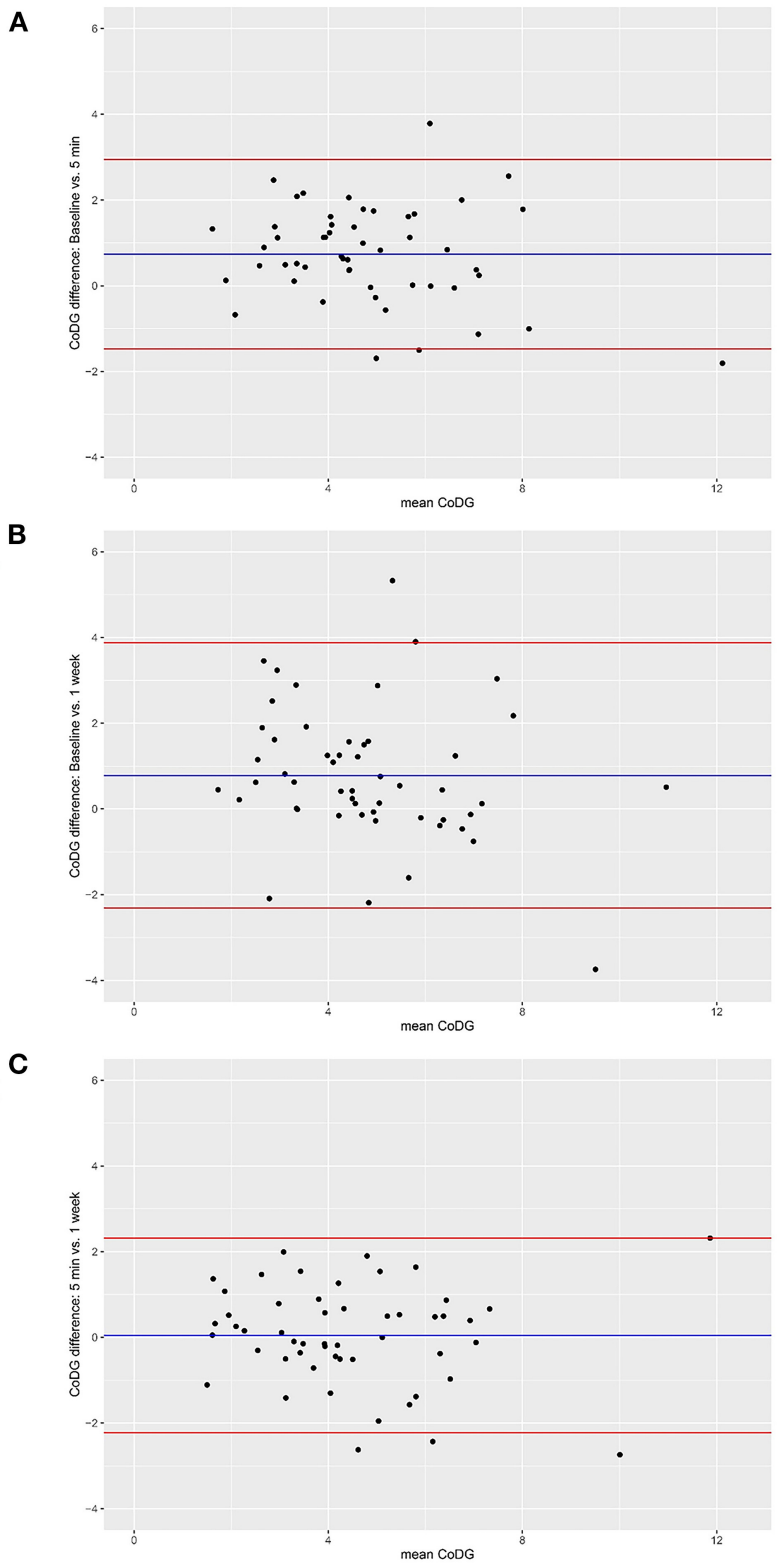
Bland–Altman plots for comparisons between baseline and after 5 min **(A)**, between baseline and after 1 week **(B)**, and between sessions after 5 min and 1 week **(C)**. Red lines depict 95% CI, blue line depicts grand mean. The *Y*-axis of the Bland–Altman plot represents the difference between paired measurements (intra-individual variation) while the *X*-axis represents the mean of the paired measurements (inter-individual variation).

The CoDG ranged between 1.74° and 11.22° (mean: 5.21°) at baseline; between 0.95° and 13.02° (mean: 4.47°) after 5 min and between 0.95° and 11.38° (mean: 4.43°) after 1 week. Individual CoDGs are depicted in Figure 2. Despite the slight decrease of the mean CoDG after 5 min, intra-class correlations revealed high consistency over all three sessions (ICC = 0.763, 95% CI [0.629, 0.855], *p* < 0.001) and similarly high consistency when comparing baseline to the session after 5 min (ICC = 0.783, 95% CI [0.498, 0.894], *p* < 0.001), or after 1 week (ICC = 0.649, 95% CI [0.402, 0.796], *p* < 0.001). The highest consistency was found between the session after 5 min and 1 week (ICC = 0.855, 95% CI [0.760, 0.914], *p* < 0.001). The intra- and inter-individual differences of CoDG are visualized in the Bland–Altman plots (Figures 3A–C).

Inspection of the Bland–Altman plots reveals high inter-individual variability and also some intra-individual variability. Inter-individual variability is depicted by a wide spread of the data points on the x-axis, ranging from 1.51° to 12.12°. Intra-individual variability is illustrated by the spread of the data points on the *y*-axis (between −1.81 and 3.79 for the comparison between baseline and 5 min; between −3.74 and 5.33 for the comparison between baseline and 1 week; and between −2.74 and 2.32 for the comparison between 5 min and 1 week). However, the vast majority lie within the 95% CI, in all three comparisons. There was no systematic change in CoDG between any two sessions (visualized by the grand mean line lying close to zero in all three plots).

### Relation Between CoDG and Questionnaires

The CoDG correlated neither with the AQ-k (*r* = 0.036, *p* = 0.802) nor with the SPIN (*r* = −0.120, *p* = 0.400), suggesting no relation between autistic or social anxiety traits and the CoDG in the present sample.

The rmANOVA revealed no main effect of session *F*_(1.927, 88.64)_ = 2.982, *MSE* = 0.846, *p* = 0.058, $\eta_{p}^{2}$ = 0.061. However, Bonferroni-corrected pairwise comparisons revealed that the CoDG in the baseline session was significantly wider than after 5 min (*p* = 0.001) and 1 week (*p* < 0.001). The CoDG in the sessions carried out after 5 min and after 1 week did not differ from each other (*p* = 1.000). Participant sex had no significant effect on the CoDG (*p* = 0.877), neither did age (*p* = 0.834) or values of AQ-k (*p* = 0.271) or SPIN (*p* = 0.064) affect the CoDG.

## Brief Discussion

The results of Experiment 1 confirmed previous findings that the CoDG varies substantially between individuals (e.g., Gianotti et al., 2018). In addition, and more interestingly, our data suggest that intra-individually, the CoDG is stable across different time points: We found good to very good consistency as measured with intra-class coefficients. This suggests that the CoDG can be seen as a stable individual trait, with considerable inter-individual variation.

In contrast to previous studies, we found no relation between the CoDG and traits of social anxiety (SAD) or autism (ASD), as measured with brief screening questionnaires (SPIN and AQ-k). We note, however, that the present study was not designed to test the hypothesis that traits of SAD or ASD are related to the CoDG. We recruited healthy participants where we expect the inter-individual variability of SAD and ASD traits to be limited, occluding possible relationships with CoDG.

Closer inspection of the data suggest a relative narrowing between the CoDG at baseline and after 5 min. In contrast, the width of the CoDG was virtually unchanged between the measurement after 5 min and after 1 week. Possibly, this was due to a practice effect as a consequence of the repeated measures design. We hence conducted a second experiment where the measurement of the CoDG was repeated after 1 week only. If the CoDG decreases with practice, we would expect a similar decrease in the width of the CoDG after 1 week as we saw after 5 min.

In Experiment one, participants completed the experiment three times, at baseline, after 5 min and after 1 week. In all repetitions, we found that the CoDG was highly consistent. It is unclear whether the CoDG would also be stable if the measurement was repeated after 1 week only, without an intermediate session after 5 min. In Experiment 2, we repeated the CoDG measurement after 1 week only.

## Experiment 2: CoDG at Baseline and After 1 Week

### Methods

#### Participants

A total of 60 participants (30 men, 30 women) aged between 19 and 54 years (*M* = 25.3, *SD* = 9.71) volunteered to take part in this study. All participants had normal or corrected to normal vision. Five participants were excluded from the analysis due to inconsistent responses recorded during the task, which precluded the estimation of Cone of Direct Gaze (CoDG). All participants gave written informed consent and were informed of their right to discontinue participation at any time. Data were collected in a single wave and then analyzed (no analyses were calculated before all participants were tested). Each participant was tested twice, once at baseline (T0) and once 1 week later (T1).

#### Stimuli

The stimuli were the same as in Experiment 1.

#### Task and Procedure

The identical task and procedure was used as in Experiment 1, except that there were only two test sessions, separated by 1 week. At the end of the second test session participants filled in the short version of the *Autism Spectrum Quotient* (Baron-Cohen et al., 2001; German version by Freitag et al., 2007) and the *Social Phobia Inventory* (Connor et al., 2000).

#### Statistical Analyses

Data were analyzed in the same way as in Experiment 1.

## Results

**Figure 4:**
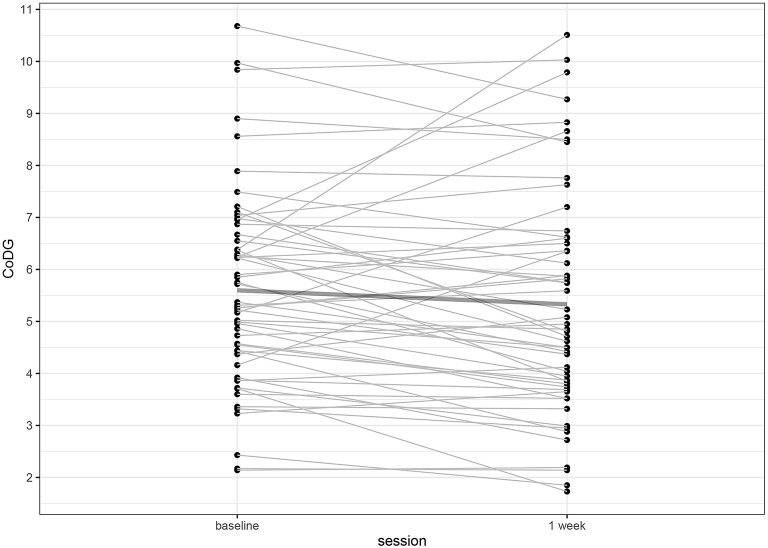
Individual CoDG in angular degrees, separately for baseline, and after 1 week. The bold line depicts the mean CoDG.

**Figure 5:**
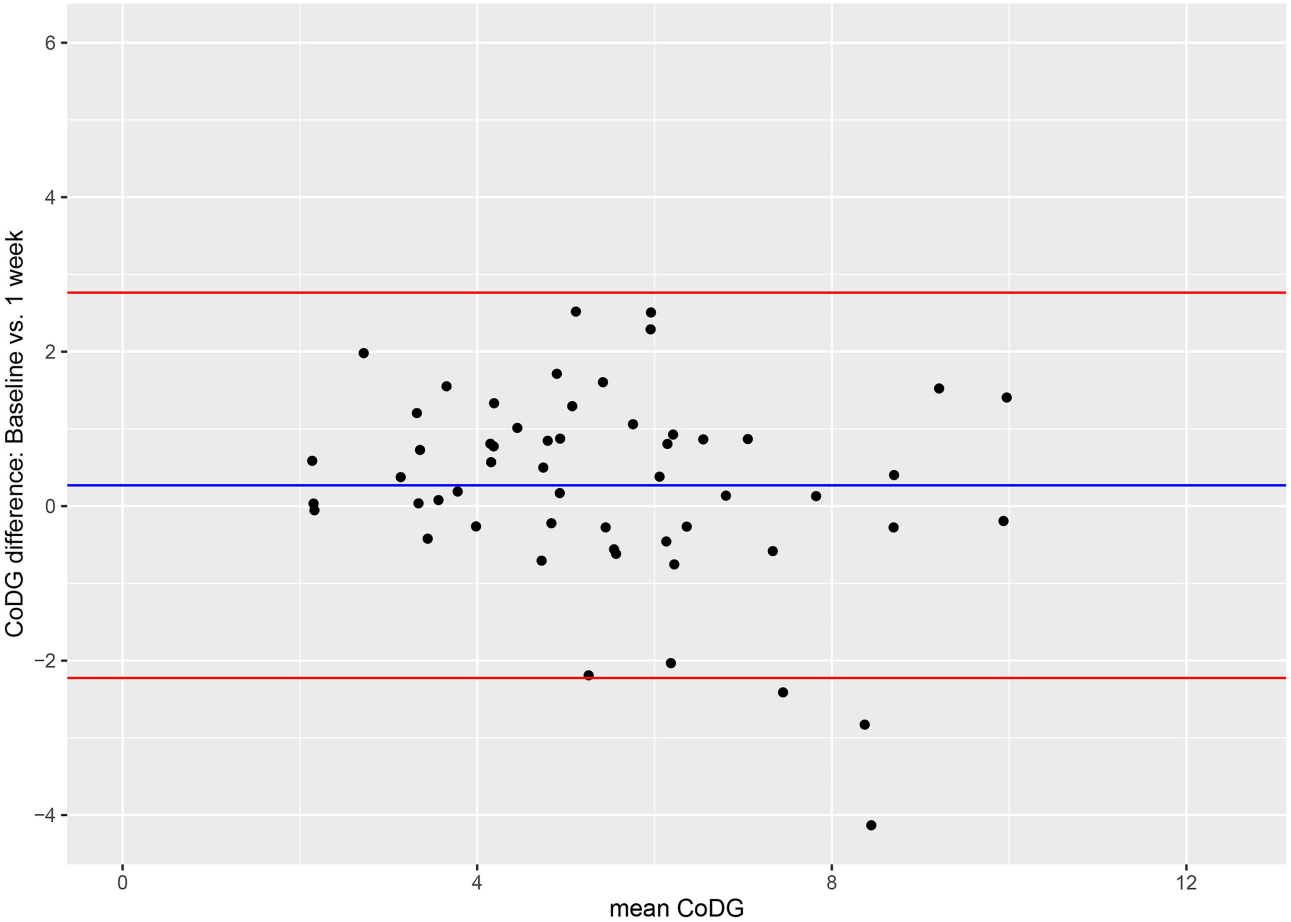
Bland–Altman plots for comparison between baseline and after 1 week. Red lines depict 95% CI, blue line depicts grand mean. The *Y-*axis of the Bland–Altman plot represents the difference between paired measurements (intra-individual variation) while the *X-*axis represents the mean of the paired measurements (inter-individual variation).

The CoDG ranged between 2.14° and 10.68° (mean: 5.60°) at baseline and between 1.73° and 10.51° (mean: 5.33°) after 1 week. Individual CoDGs are depicted in Figure 4. Intra-class correlations revealed high consistency (ICC = 0.800, 95% CI [0.680, 0.878], *p* < 0.001). The intra- and inter-individual differences of CoDG are visualized in the Bland–Altman plot (Figure 5).

Inspection of the Bland–Altman plot reveals high inter-individual variability depicted by a wide spread of the data points on the *x*-axis (between 2.14° and 9.97°), and also some intra-individual variability (illustrated by the fact that on the y-axis the data points spread between −4.13 and 2.52). However, the majority lie within the 95% CI. There was no systematic change in CoDG between baseline and after 1 week (visualized by the grand mean line lying close to zero).

### Relation Between CoDG and Questionnaires

The CoDG correlated neither with the AQ-k (*r* = −0.005, *p* = 0.970) nor with the SPIN (*r* = −0.127, *p* = 0.355), suggesting no relation between autistic or social anxiety traits and the CoDG in the present sample.

The rmANOVA revealed no main effect of session *F*_(1, 50)_ = 1.206, *MSE* = 0.830, *p* = 0.277, $\eta_{p}^{2}$ = 0.024. Participant sex had no significant effect on the CoDG (*p* = 0.165), neither did participant age (*p* = 0.154) or values of AQ-k (*p* = 0.762) or SPIN (*p* = 0.751) affect the CoDG.

## Brief Discussion

Consistent with Experiment 1 and with previous work, we found large inter-individual variability of the CoDG. Again as in Experiment 1, we found only very moderate intra-individual variability of the CoDG. The widths of the CoDG in the two sessions (at baseline and after 1 week) were very consistent, thus corroborating findings from Experiment 1 that the CoDG is stable over time. Further, we again found no relation between measures of SAD and ASD in this cohort of participants. As in Experiment 1, we recruited our participants from the general public, characterized by relatively low and homogenous levels of SAD and ASD, potentially concealing the interrelationship between CoDG and SAD or ASD measures found in previous studies.

Interestingly, there was no significant narrowing of the CoDG between baseline measurement and when repeated after 1 week. This is in contrast to Experiment 1, where the the CoDG narrowed between baseline and after 5 min. The findings of Experiment two underline the robustness of the CoDG as expressed by a good absolute intra-individual agreement between repeated measurements.

## General Discussion

The cone of direct gaze (CoDG) refers to the range of gaze angles that an observer judges as being directed at them. Recent studies revealed remarkable individual differences in the range of gaze angles that are accepted as making eye contact (e.g., Ewbank et al., 2009; Gamer et al., 2011; Schulze et al., 2013; Harbort et al., 2017; Gianotti et al., 2018). We aimed at establishing how stable an individual's CoDG is over time. Employing an established forced-choice judgment task to measure the CoDG, we found highly consistent measures of CoDG when repeated after 5 min and/or after 1 week, suggesting that the CoDG is a stable measure that can be considered as a trait measure.

Interestingly, the time span between repetitions of the task had only limited bearing on the width of the CoDG. We found a high absolute agreement of the CoDG between baseline and after 5 min. When repeated a second time after 1 week, the CoDG was again highly consistent. In Experiment 2, the CoDG measurement was not repeated after 5 min, but instead only after 1 week. Here the CoDG measurements were also highly consistent. Our findings thus suggest that the CoDG is a stable measure over time, irrespective of the amount of time passed between repetitions, and irrespective of the number of repetitions.

Despite an overall good absolute agreement between repeated measurements, we observed a narrowing of the CoDG in Experiment 1 between the baseline measurement and after 5 min. This may hint toward a familiarity effect, which unfolds specifically after short-term repetitions. Interestingly, the CoDG did not significantly narrow when repeated after 1 week intervals. Taken together, this underlines the robustness of the CoDG as expressed by a good absolute intra-individual agreement between repeated measurements.

Consistent with previous studies, we found remarkable inter-individual differences in the CoDG, which were much larger than the intra-individual variations (cf. Bland–Altman plots). Individual differences in the width of the CoDG have been associated with clinical conditions such as social anxiety or autism. These studies consistently reported that individuals with social anxiety disorder (SAD) accept a wider range of gaze lines to be directed at them (e.g., Gamer et al., 2011; Jun et al., 2013). Autism spectrum disorder (ASD) has been related to narrower CoDG, at least in males (Matsuyoshi et al., 2014). In the present study, the CoDG was neither related to ASD (as measured with the AQ-k) nor was it related to symptoms of social anxiety (as measured with the SPIN). The SPIN is a questionnaire developed for screening and measuring severity of social anxiety disorder and the AQ-k aims to investigate whether adults of average intelligence have symptoms of autism spectrum conditions. We note that the present study was not designed to test the hypothesis whether traits of SAD or ASD are related to the CoDG. The participants in the present experiments were sampled from the general public and our sample contained no clinically diagnosed patients. We thus expect the inter-individual variability of SAD and ASD traits to be limited in the present sample, occluding possible relationships with CoDG. Both the SPIN and the AQ-k may not be sensitive enough to detect fine-tuned individual differences in the healthy population. Also, especially the expression of SAD traits in the CoDG might need a more ecologically valid situation than the laboratory context of the present study. Future exploration with this type of task needs to assess those relationships with more realistic designs.

Studies relating higher SAD scores with wider CoDG have implied that the CoDG could be seen as a marker of social anxiety and could be used either as a diagnostic measure or as a measure for the efficacy of a therapy (e.g., Jun et al., 2013). A necessary precondition for this is that the CoDG is a relatively stable measure that does not randomly vary from one ascertainment to another. Our findings suggest that this is the case. We repeated the measurement of the CoDG under highly standardized conditions over two or three time points and found high test–retest reliability using ICCs, demonstrating high absolute agreement over time. These findings suggest that people have highly consistent CoDG over time.

## Data Availability Statement

The original contributions presented in the study are included in the article/Supplementary Material, further inquiries can be directed to the corresponding author/s.

## Ethics Statement

The studies involving human participants were reviewed and approved by Ethics Committee of the Faculty of Human Sciences, University of Bern. The patients/participants provided their written informed consent to participate in this study.

## Author Contributions

JL, BS, TB, and DK developed the study concept and contributed to the design. JL and BS collected the data. JL analyzed the data with help of BS and TB and wrote the first draft of the manuscript. All authors helped to edit and revise the manuscript and approved the final submitted version of the manuscript.

## Conflict of Interest

The authors declare that the research was conducted in the absence of any commercial or financial relationships that could be construed as a potential conflict of interest.
